# Exploiting algal NADPH oxidase for biophotovoltaic energy

**DOI:** 10.1111/pbi.12332

**Published:** 2015-01-29

**Authors:** Alexander Anderson, Anuphon Laohavisit, Ian K. Blaby, Paolo Bombelli, Christopher J. Howe, Sabeeha S. Merchant, Julia M. Davies, Alison G. Smith

**Affiliations:** ^1^ Department of Plant SciencesUniversity of CambridgeCambridgeUK; ^2^ Department of Chemistry and BiochemistryUCLALos AngelesCAUSA; ^3^ Department of BiochemistryUniversity of CambridgeCambridgeUK; ^4^ Institute for Genomics and ProteomicsUCLALos AngelesCAUSA; ^5^ Present address: Department of Physiology, Development and NeuroscienceUniversity of CambridgeCambridgeCB2 3EGUK; ^6^ Present address: RIKEN Centre for Sustainable Resource Sciences1‐7‐22 SuehiroTsurumiYokohamaKanagawa230‐0045Japan

**Keywords:** alga, biophotovoltaic, *Chlamydomonas*, energy, NADPH oxidase

## Abstract

Photosynthetic microbes exhibit light‐dependent electron export across the cell membrane, which can generate electricity in biological photovoltaic (BPV) devices. How electrons are exported remains to be determined; the identification of mechanisms would help selection or generation of photosynthetic microbes capable of enhanced electrical output. We show that plasma membrane NADPH oxidase activity is a significant component of light‐dependent generation of electricity by the unicellular green alga *Chlamydomonas reinhardtii*. NADPH oxidases export electrons across the plasma membrane to form superoxide anion from oxygen. The *C. reinhardtii* mutant lacking the NADPH oxidase encoded by *RBO1* is impaired in both extracellular superoxide anion production and current generation in a BPV device. Complementation with the wild‐type gene restores both capacities, demonstrating the role of the enzyme in electron export. Monitoring light‐dependent extracellular superoxide production with a colorimetric assay is shown to be an effective way of screening for electrogenic potential of candidate algal strains. The results show that algal NADPH oxidases are important for superoxide anion production and open avenues for optimizing the biological component of these devices.

## Introduction

Renewable power sources such as wind farms, solar panels or hydro turbines are now in relatively widespread use, yet the feasibility of these to replace fossil fuels is uncertain due to their higher cost and lower energy density (Cho, 2010). Therefore, in the past decade, there has been a considerable shift in research focus into harvesting energy from high‐biomass biological materials. However, considerable areas of arable land are required to sustain biofuel production, which is at odds with food production (Jones and Mayfield, 2012). As a result, the use of algae as novel biofuel sources has rapidly gained popularity in recent years. Individually, algae are simple photosynthetic organisms, yet together constitute over 300 000 species with great evolutionary diversity. Their rapid growth, high‐biomass yields and ability to thrive in freshwater, salt water and wastewater could be highly advantageous for biofuel production. They do not compete for space with commercial crop species (Scott *et al*., 2010), and their photosynthetic capacity is excellent, with energy efficiency levels on a par with land plants (Larkum, 2010). At the level of the primary light reaction, when the energy of light is used to do photosynthetic work in terms of water photolysis, up to 37% efficiency has been estimated (Zhu *et al*., 2010). Total worldwide energy consumption is around 15 terawatts per year, so with over 85 000 terawatts of solar energy reaching the Earth annually, this represents a lucrative source of renewable power even if only some of it could be harnessed (Jones and Mayfield, 2012).

**Figure 1:**
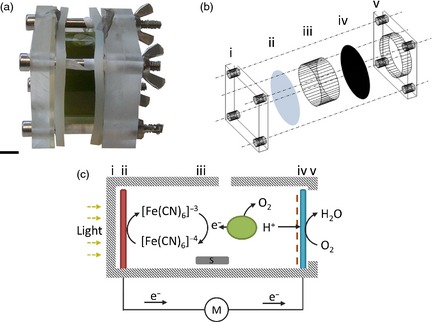
BPV design and function. (a) Image of assembled BPV device containing *Chlamydomonas reinhardtii* cells in the anodic chamber. Scale bar, 1 cm. (b) Exploded schematic of the anodic chamber. The front clamp (i) permits light to enter *via* the anode (ii) into the algal chamber (iii) and the back clamp (v) has a cut‐out to allow oxygen contact with the cathode (iv). (c) Principles of operation of a BPV device. Upon illumination, cells (green ovoid) within the algal chamber release electrons (e^−^) which reduce extracellular ferricyanide ([Fe(CN)_6_]^4−^) to ferrocyanide ([Fe(CN)_6_]^3−^). Ferrocyanide shuttles electrons to the anode (red rectangle, ii) into the external circuit *via* an external resister and multimeter (V) through to the cathode (blue rectangle, iv). Simultaneously, protons (H^+^) diffuse from the chamber *via* a dialysis and Nafion membrane (dotted line) to the cathode combining with electrons and oxygen to form water. Cells are kept in suspension by a magnetic stirrer (S) and evaporation limited by sealing chamber (B). Wires are connected *via* crocodile clips to two stainless steel strips. Numbers above selected components in (b) correspond to those shown in (c).

Algae also have the potential for direct production of electricity, in addition to providing combustible biomass for its generation. This is increasingly relevant as the quest for renewable energy has included the development of microbial fuel cells that utilize biological electron export processes to generate electricity (Rosenbaum *et al*., 2010). Using photosynthetic microbes such as microalgae in these fuel cells reduces the need for a supply of fixed carbon and effectively creates a biological solar panel, often termed biological photovoltaic (BPV) devices (Bombelli *et al*., 2011; Samsonoff *et al*., 2014; Figure 1a and b). Light energy is captured by the photosynthetic apparatus, leading to electron export across the cell membrane (Bradley *et al*., 2012). Electrons can then be captured by a soluble electron carrier in the aqueous medium for delivery to the anode and current generation (Bombelli *et al*., 2011; Figure 1c). Alternatively, electrons can be delivered directly to the anode by biofilms growing on its surface (McCormick *et al*., 2011). A limiting factor is cellular electron export and so identifying the electron transport proteins that contribute power output in the devices remains a fundamental goal in the development of these biological solar cells.

**Figure 2:**
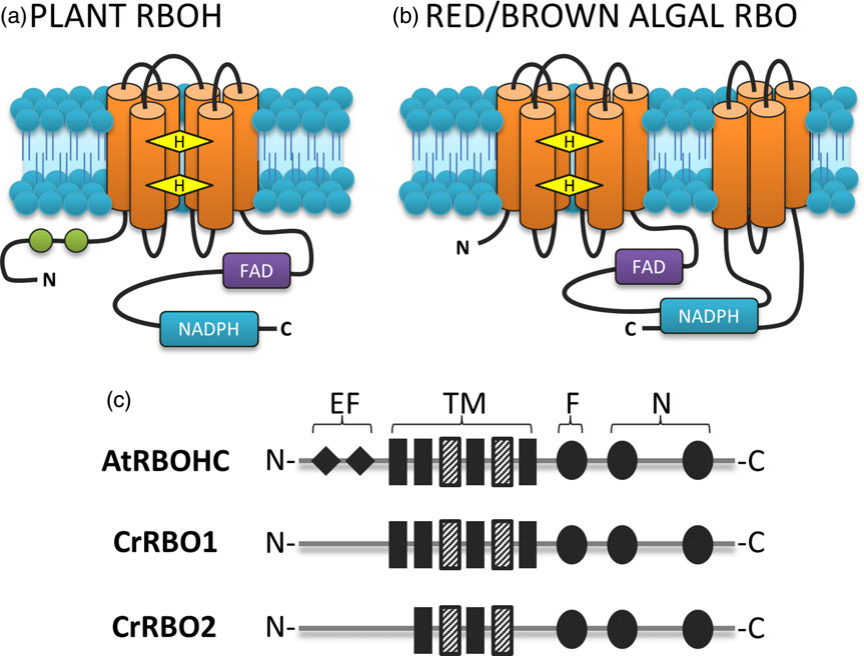
Comparison of plant and algal NADPH oxidase structures. (a) Plant NADPH oxidase (RBOH) structure based on sequence data from *Arabidopsis thaliana* and other plant NOX described in Torres *et al*. (1998) and Kawahara *et al*. (2007). (b) Algal RBO structure based on sequence data from *Chondrus crispus, Cyanidioschyzon merolae, Porphyra Yezoensis* and *Phaeodactylum tricornutum* described in Hervé *et al*. (2005). NOX in these algae have an additional four transmembrane domains in between the NADPH‐binding domain. These are absent from the *Chlamydomonas* RBO. (c) Schematic of RBO proteins CrRBO1 and CrRBO2 compared to *Arabidopsis thaliana* AtRBOHC. Abbreviations: EF, EF hand; F, FAD‐binding domain; N, NADPH‐binding domains; TM, transmembrane domain. Dashed TM domains indicate the presence of conserved haem‐binding histidine residues. Proteins are orientated with the cytosolic side at bottom of image. Orange cylinders represent transmembrane domains, green circles represent EF hands, and yellow diamonds represent haem molecules. Binding regions for FAD and NADPH are highlighted.

Plasma membrane NADPH oxidases (NOX) are found in animals, plants and algae. They are encoded in animals by *NOX* genes and in plants by respiratory burst oxidase homologue (*RBOH*) genes (named from homology with the animal respiratory burst NOX; Figure 2). They catalyse the transfer of electrons from cytosolic NADPH to extracellular oxygen, so generating extracellular superoxide anion ($O_{2}^{-}$; Sagi *et al*., 2004). The ability of NOX to generate an electrical current was demonstrated by Schrenzel *et al*. (1998) by whole‐cell patch clamping of human phagocytes (in which NOX are the main transmembrane redox component). Current was dependent on the presence of intracellular NADPH and was diminished by the widely used NOX inhibitor diphenylene iodonium chloride (DPI). Additionally, mutated cells lacking a functional NOX showed no detectable current, demonstrating the importance of NOX as an electrogenic component (Schrenzel *et al*., 1998). Subsequently, power output of a fuel cell containing human neutrophils was shown to be dependent on NOX activity (Sakai *et al*., 2009). The presence of DPI or inhibitors of NOX protein assembly inhibited power output. These enzymes in photosynthetic microbes are therefore targets for development in the improvement of BPV output.

In plants, mutant studies have shown that NOX are involved in immunity, development and stress responses (Foreman *et al*., 2003; Kadota *et al*., 2014; Lee *et al*., 2013). In contrast to the research on animal and plant NOX, the research on algal NOX (encoded by *RBO* genes; Figure 2) is much less advanced. Extracellular superoxide production has been recorded in many species of red, green and brown algae and diatoms (Marshall *et al*., 2005), suggesting that algal NOX may be widespread. They have been proposed to be involved in growth, stress and immune responses (reviewed by Anderson *et al*., 2011). Production can be inhibited by DPI, suggesting that this is a useful tool in delineating NOX activity (Bouarab, 1999; Küpper *et al*., 2001; Ross *et al*., 2005; Coelho *et al*., 2008; Pérez‐Pérez *et al*., 2012). In the unicellular raphidophyte *Chattonella marina*, superoxide production is at the plasma membrane and stimulated by light (Kim *et al*., 2004, 2007) implying that reductants for superoxide generation are sourced from photosynthesis. Molecular data for the *Chondrus crispus* NOX showed that typical human NOX2 features are highly conserved, including the substrate‐binding sites, haem‐binding histidine residues and transmembrane domains. There is substantial evidence for NOX in algae, and therefore, they may function as plasma membrane electron transporters for use in BPV devices (Bombelli *et al*., 2011).

The unicellular green alga *Chlamydomonas reinhardtii* generates extracellular superoxide (Hema *et al*., 2007; Hemschemeier *et al*., 2013; Pérez‐Pérez *et al*., 2012). Its two *RBO* genes are predicted to encode NOX with the conserved NADPH‐binding domains and the transmembrane domains with haem‐liganding histidine residues that are essential for electron transport (Hervé *et al*., 2005; Anderson *et al*., 2011; Figure 2). However, at present, the contribution of the two RBO proteins to electron export and extracellular $O_{2}^{-}$ production is unknown. We therefore tested the hypothesis that an algal NOX could contribute to power output in a BPV device by comparing *C. reinhardtii* strain *cw15* (a standard laboratory strain in which the cell wall is greatly reduced) and its *sta6rbo1* mutant (Li *et al*., 2010), in which *STA6* is mutated and *RBO1* is deleted (Blaby *et al*., 2013; Table 1). The results show that RBO1 is indeed responsible for extracellular superoxide anion production and that this generates a significant part of the current measured in the BPV device. *RBO* expression in *C. reinhardtii* can be maintained even under nutrient limitation (Allen *et al*., 2007) and *RBOL2* expression increases under anaerobiosis (Hemschemeier *et al*., 2013). As algae produce $O_{2}^{-}$ even under adverse conditions including as a response to predation or infection (Hervé *et al*., 2005; Hema *et al*., 2007; Kim *et al*., 2007;  Küpper *et al*., 2001; Anderson *et al*., 2011), these photosynthetic microbes would be a robust, exploitable and renewable resource for application in biological solar cells.

**Table 1:** Summary of algal strains used in this study

Strain	Description
*cw92*	Cell wall‐deficient, WT for STA6 and RBO1
*cw15*	Cell wall‐deficient, WT for STA6 and RBO1
*sta6rbo1*	*cw15* carrying mutated *STArchless6* and with a deleted *RBO1*
*sta6rbo1(RBO1)*	*cw15* carrying mutated *STArchless6* and with a deleted *RBO1* but complemented for *RBO1*
*sta6rbo1(STA6)*	*cw15* carrying mutated *STArchless6* and with a deleted *RBO1* but complemented for *STA6*

## Results

### Production of extracellular superoxide anion is light‐dependent and requires RBO1

**Figure 3:**
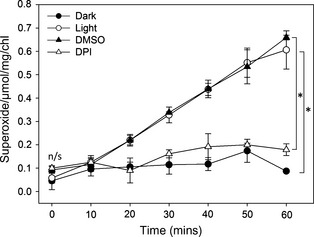
*Chlamydomonas reinhardtii* extracellular superoxide anion production is light‐dependent and DPI sensitive. Time course of $O_{2}^{-}$ production by mid‐logarithmic cells of the *Chlamydomonas reinhardtii* strain *cw92*, determined using XTT reduction. Mean ± SEM $O_{2}^{-}$ production was prevented by dark incubation and inhibited in the light by DPI (20 μm) as a NOX inhibitor. The equivalent dimethylsulphoxide (DMSO) concentration was used as the DPI solvent control (0.0075% v/v). Asterisks denote significant difference (*P *<* *0.05) from control (Student's *t*‐test; n/s, no significance; *n *=* *3).

**Figure 4:**
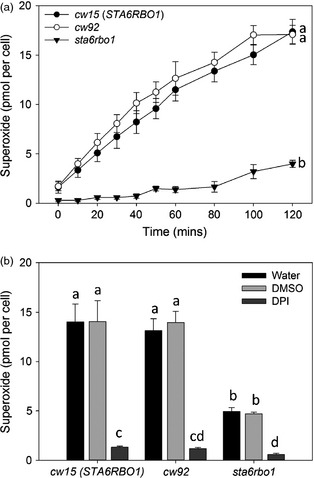
*Chlamydomonas reinhardtii* extracellular superoxide anion production requires RBO1. (a) Time course of $O_{2}^{-}$ production by mid‐logarithmic cells of the *Chlamydomonas reinhardtii c*ell *w*all‐deficient strains *cw15*,* cw92* (both *STA6RBO1*) and the *RBO1‐*deficient *sta6rbo1* mutant, determined using XTT reduction. Levels of $O_{2}^{-}$ production were indistinguishable between both cell wall‐less strains *cw15* and *cw92*. Different letters denote significant differences (one‐way ANOVA, *P *<* *0.05). Data are mean ± SEM (*n = 3*). (b) DPI (20 μm) inhibited light‐dependent $O_{2}^{-}$ production by all strains. Values are for 120 min. (one‐way ANOVA, *P *<* *0.05; mean ± SEM, *n = 3*). There was no effect of equivalent concentration (v/v) of DMSO as the solvent for DPI. Different letters denote significant differences (one‐way ANOVA, *P *<* *0.05). Data are mean ± SEM (*n = 3*).

We first confirmed that extracellular superoxide anion production could be detected from *C. reinhardtii* by assaying the reduction of cell‐impermeable XTT (2,3‐*bis*‐(2‐methoxy‐4‐nitro‐5‐sulfophenyl)‐2*H*‐tetrazolium‐5‐carboxanilide (Sutherland and Learmonth, 1997). The cell wall‐deficient strain *cw92*, which is wild type for both *RBO1* and *STA6* (*STArchless6* encoding the small subunit of ADP‐Glc pyrophosphorylase; Table 1), was grown to mid‐logarithmic phase. These cells supported light‐dependent production of extracellular superoxide anion that was significantly inhibited by DPI, indicating NOX activity (Figure 3). Extracellular superoxide anion production by c*w92* was indistinguishable from that of mid‐logarithmic *cw15*, the cell wall‐deficient *STA6RBO1* strain from which the *sta6rbo1* mutant is derived (Figure 4; Table 1). Both *cw15* and *cw92* were derived from mutagenesis of the WT 137c (Davies and Plaskitt, 1971; Pröschold *et al*., 2005). The genetic basis for the *cw* mutation remains unknown, but *cw92* contains a glycoprotein in its residual cell wall that is absent from *cw15* (Voigt *et al*., 1996). Light‐dependent extracellular $O_{2}^{-}$ production by *cw15* was significantly inhibited by DPI, indicating production by NOX. Critically, light‐dependent production by *sta6rbo1* was significantly impaired even without DPI addition, which suggests that RBO1 was responsible for the majority of $O_{2}^{-}$ production (Figure 4). The residual $O_{2}^{-}$ production by *sta6rbo1* that was DPI‐sensitive could be due to the continued low expression of *RBO2* (Blaby *et al*., 2013). The DPI‐insensitive $O_{2}^{-}$ production shown by all three strains suggests that alternative cell membrane redox enzymes are still in operation.

### Complementation with *RBO1* restores $O_{2}^{-}$ production

**Figure 5:**
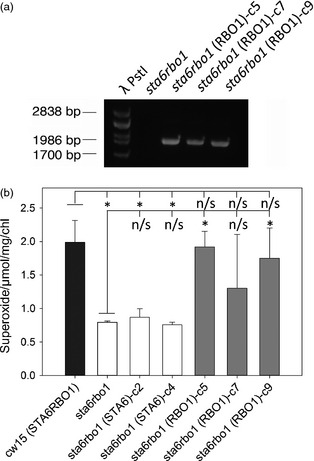
Superoxide anion production is restored by *RBO1*. (a) Validation of pSL18_RBO1 insertion into *sta6rbo1* (CC‐4348) genome. To verify integration of the *RBO1*‐containing construct, PCRs were performed using primers specific to the plasmid. The predicted amplicon size is 1979 bp. (b) Mean ± SEM $O_{2}^{-}$ production in light (120 min) from *cw15*,* RBO1*‐deficient *sta6*, two lines of *sta6rbo1(STA6)* (Blaby *et al*., 2013) and the three *RBO1*‐complemented lines shown in (a), determined using XTT (*n *≤* *5). Only the presence of *RBO1* was effective in restoring $O_{2}^{-}$ production. Asterisks denote significant difference (*P *<* *0.05) from indicated control (Student's *t*‐test; n/s, no significance).

If RBO1 were the source of extracellular $O_{2}^{-}$ production then it follows that complementing the *sta6rbo1* mutant with *RBO1* would restore production. To test this, the *sta6rbo1* mutant was complemented with a synthetic gene construct containing the complete *RBO1* gene model (Table 1). Three independently transformed strains were selected by antibiotic resistance and confirmed for RBO1 by PCR (Figure 5a). These were compared for light‐dependent $O_{2}^{-}$ production against two *sta6rbo1* strains that had previously been transformed to express only *STA6* and so retained loss of RBO1 function (Blaby *et al*., 2013; Table 1). Only complementation with *RBO1* restored $O_{2}^{-}$ production to levels comparable to (and not significantly different from) that of the *cw15* STA6RBO1 parental strain (Figure 5b). Complementation with *STA6* did not increase production above that of the *sta6rbo1* mutant (Figure 5b). These data show that $O_{2}^{-}$ production is indeed *RBO1* dependent.

### *RBO1* contributes to current output in the BPV device

**Figure 6:**
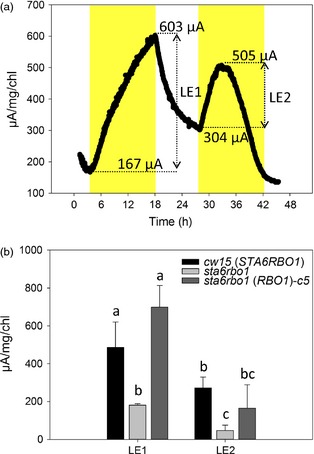
*RBO1*‐dependent current from *Chlamydomonas reinhardtii*. (a) Example current from the *cw15* parental strain in the BPV device. Yellow indicates light application; LE1 and LE2 denote the first and second light effect (LE) current. (b) Mean ± SEM of LE1 and LE2 currents from *Chlamydomonas reinhardtii* cultures (*n *=* *3–4)*,* standardized to chlorophyll (Chl) content. *sta6rbo1(RBO1)*‐*c5* is one of the three strains complemented with *RBO1* and exhibiting normal $O_{2}^{-}$ production (Figure 5b). Letters denote significant differences as determined by one‐way ANOVA (*P *<* *0.05).

Having shown that *RBO1* expression results in extracellular $O_{2}^{-}$ production, which is in turn the result of electron extrusion across the plasma membrane, we tested for a relationship between this activity and an ability to generate current in the BPV device (Figure 1). A representative current output from mid‐logarithmic *cw15* (wild type for *STA6* and *RBO1*) is shown in Figure 6a. A first application of light caused current output in the BPV device for over 12 h (Light Effect 1, LE1; Figure 6a). The return to darkness saw a decline in current, and a second application of light (LE2) was less effective in promoting current generation (Figure 6a). The reason(s) for this lower output in LE2 is not yet known. The addition of DPI to the BPV device impaired electrode function, so it was not possible to demonstrate that *cw15* treated with DPI phenocopied *sta6rbo1* as an additional line of evidence for NOX‐dependent current generation.

Light‐dependent current output was significantly impaired in the *sta6rbo1* mutant for both LE1 and LE2. Output in LE1 was fully restored by complementation with *RBO1* (Figure 6b). This was shown using the *sta6rbo1(RBO1)‐*c5 strain in which extracellular $O_{2}^{-}$ production (XTT assay) was equal to that of the *cw15* parental strain (Figure 5b). As in the parental strain, the current generation was lower on a second light exposure (LE2). Overall, significant components of both extracellular $O_{2}^{-}$ production and light‐dependent current output require *RBO1*.

## Discussion

It is clear that RBO1 makes a significant contribution to light‐dependent current generation in the BPV device, and the overall current output from *C. reinhardtii* is promising. From Figure 6b, up to approximately 800 μA/mgChl can be generated, which equals 800 μC/mgChl/s. An AAA zinc–carbon battery could have a nominal capacity of 500–700 mAh. A battery with a 650 mAh capacity holds 2340 C. A litre of *Chlamydomonas* culture with a chlorophyll concentration of 15 μg/mL would contain 15 mg of chlorophyll. It could generate 12 mC/s (800 μC/mgChl/s × 15) and would therefore take 2.25 days to generate the charge held by the battery (2340 C/0.012 C/s = 195 000 s = 2.25 days). Whether the contribution to output by RBO1 can be increased now needs to be examined. Additionally, the electron export mechanisms that were evident in the absence of RBO1 should now be identified.

Selecting algae for both biofilm formation and high NOX activity is a realistic goal. Extracellular superoxide anion production is widespread and has already been recorded from other green algae such as *Dasycladus vermicularis* (Ross *et al*., 2005), *Ulva pertusa* (Goh *et al*., 2010), *Ulva compressa* (Gonzalez *et al*., 2010), the brown alga *Laminaria digitata* (Küpper *et al*., 2001), the red alga *Chondrus crispus* (Bouarab, 1999) and red intertidal algae (Liu and Pang, 2010). In the unicellular raphidophyte *Chattonella marina*, superoxide production is essential for growth. The level of extracellular superoxide anion peaks during the exponential growth phase, and growth is inhibited in the presence of superoxide dismutase (Oda *et al*., 1995). Additionally, superoxide levels were stimulated by light (Kim *et al*., 2004) implying reductants for superoxide generation by *C. marina* are sourced from photosynthesis (Marshall *et al*., 2005). Although it has been speculated that NOX could make a contribution to production, there has been no genetic evidence up until now and the findings here on *Chlamydomonas RBO1* will enable future studies on the physiological roles and regulation of algal NOX that could be exploited for power output. Undoubtedly however, there will be other limiting factors besides electron export to overcome to ensure growth and productivity of algal cultures.

Assaying $O_{2}^{-}$ production with XTT affords a more rapid assessment of NOX activity than measuring current and could be used for screening. The widespread presence of NOX genes in freshwater and marine algae makes these organisms an attractive prospect for deployment in BPV devices. We envisage niche, off‐grid applications in which small‐scale algal production could power, for example, mobile phones or LED lights.

## Experimental procedures

### Algal strains and growth conditions

*Chlamydomonas reinhardtii* strains *cw15* (CC‐1883) and *cw92* (CC‐503) were gifts from Dr Saul Purton (UCL, London, UK). *sta6rbo1* (CC‐4348) and complemented strains s*ta6rbo1(STA6)*‐c2 (CC‐4565) and s*ta6rbo1(STA6)*‐c4 (CC‐4566) were from Dr. Ursula Goodenough (Washington University, St. Louis, MO). Strains are described in Table 1. They were grown axenically in Tris‐acetate phosphate (TAP) medium prepared as described (http://www.chlamy.org/methods.html). Cultures were inoculated using cells from stationary phase stock cultures (inoculum density 20 000 cells/mL) and grown under continuous light (~40 μE/m^2^/s, 21 °C, 140 rpm; INFORS HT, Reigate, Surrey, UK). Chlorophyll was extracted according to Inskeep and Bloom (1985).

### Complementation of RBO1

To restore *rbo1* function to *sta6rbo1* (CC‐4348), a synthetic gene construct containing the complete *RBO1* gene model (Genscript, Piscataway, New Jersey, USA) designed based on the v5.3 assembly of the *C. reinhardtii* genome (http://www.phytozome.net/chlamy.php) was excised from pUC57 by NdeI SpeI digest and inserted into the NdeI SpeI sites of pSL18 (Fischer and Rochaix, 2001) to generate pSL18_RBO1. pSL18_RBO1 was linearized by XhoI digestion and transformed into CC‐4348. Transformants were selected for by paromomycin resistance (5 μg/mL) and candidate colonies were PCR‐validated using the primers specific to the plasmid (5′ to 3′: forward primer ATTATGTATCAATATTGTTGCGTTCG, reverse primer GTCACCACAAACACAACGGCGAG). PCR amplicons were sequenced to confirm product.

### Superoxide measurements

Algal cells in TAP medium were collected by centrifugation (4000 *g*, 5 min), and cells were washed in buffer (Tris‐HCl, 20 mm, pH 7.0) and resuspended in buffer plus 100 μm XTT (Sigma‐Aldrich, Haverhill, Suffolk, UK). Cells were transferred to a transparent Perspex chamber (except 5b for which cells were assayed in microfuge tubes), continually mixed to ensure cell suspension and illuminated with white LED lights (OSRAM Parathom Par 16, Stockport, UK; 100 μE/m^2^/s). Samples (250 μL) were removed, cells pelleted, and the absorbance at 470 nm of the supernatant measured with a plate reader (Fluostar Optima, BMG Labtech, Aylesbury, Bucks, UK) in 96‐well flat‐bottom plates (Greiner Bio‐One, Stonehouse, South Lanarkshire, UK). Superoxide was quantified according to the extinction coefficients of Sutherland and Learmonth (1997). All experiments were conducted in the light (100 μE/m/s^2^) unless stated otherwise. In inhibitor experiments, cells were exposed to DPI or DMSO for 1 h prior to experiment and then resuspended in buffer (Tris‐HCl, 20 mm pH 7.0) with DPI/DMSO present at the same concentration used in the pretreatment.

### BPV device construction and operation

BPV devices were formed from 10‐mm‐thick transparent Perspex (anodic chamber external diameter 50 mm, internal diameter 44 mm, depth 20 mm with nominal internal volume 30.5 mL and 15.2 cm^2^ illuminated area), sealed with polydimethylsiloxane, similar to the device described in Bombelli *et al*. (2011). An 80 × 80 mm carbon–platinum cathode assembly impregnated on one side (50 × 50 mm coated area) with Nafion (Ion Power, New Castle, Delaware, USA) formed the back wall of the anodic chamber. A single layer of dialysis membrane (SnakeSkin Dialysis Tubing 10 K MCWO, Sigma‐Aldrich) was layered on to the cathode assembly. Transparent indium tin oxide (ITO, 60 Ω/sq) on plastic polyethylene terephthalate (Sigma‐Aldrich) formed the front of the anodic chamber. Electrodes were attached to the external circuit via a contact formed from a strip of stainless steel. Current was monitored via a multimeter (Model UT70B, Uni‐Trend, Hong Kong) and recorded using UT70B interface software v3.04 (Uni‐Trend, Kowloon, Hong Kong, China). A bias potential (500 mV) was applied between the anode and cathode by means of an external power box made in‐house. The device was illuminated through the ITO side by a panel of red LED bulbs (maximum emission peak 630 nm) controlled by a PSU130 power unit (LASCAR, Salisbury, UK) set at 100 μE/m^2^/s. The apparatus was completely covered by black velvet fabric to ensure complete darkness. Mid‐logarithmic algal cultures in TAP medium were loaded directly into the anodic chamber. Potassium ferricyanide (FeCN) was added to a final concentration of 1 mm, and cultures were continually mixed via a magnetic stirrer (Multistirrer 6, VELP Scientifica, Usmate (MB), Italy; Setting 4). Devices were operated at room temperature (~21 °C). Dialysis membrane was replaced for each experiment, and the other components washed with deionized water before reuse.
